# Dynamic rerouting of the carbohydrate flux is key to counteracting oxidative stress

**DOI:** 10.1186/jbiol61

**Published:** 2007-12-21

**Authors:** Markus Ralser, Mirjam M Wamelink, Axel Kowald, Birgit Gerisch, Gino Heeren, Eduard A Struys, Edda Klipp, Cornelis Jakobs, Michael Breitenbach, Hans Lehrach, Sylvia Krobitsch

**Affiliations:** 1 Max Planck Institute for Molecular Genetics, Ihnestrasse 73, 14195 Berlin, Germany; 2 Department of Clinical Chemistry, Metabolic Unit, VU University Medical Center, Amsterdam, de Boelelaan 1117, 1081 HV Amsterdam, The Netherlands; 3 Department of Cell Biology, University of Salzburg, Hellbrunnerstrasse 34, 5020 Salzburg, Austria; 4 Current address: Medical Proteome Center, Ruhr University Bochum, Universitätsstrasse 150, 44801 Bochum, Germany

## Abstract

**Background:**

Eukaryotic cells have evolved various response mechanisms to counteract the deleterious consequences of oxidative stress. Among these processes, metabolic alterations seem to play an important role.

**Results:**

We recently discovered that yeast cells with reduced activity of the key glycolytic enzyme triosephosphate isomerase exhibit an increased resistance to the thiol-oxidizing reagent diamide. Here we show that this phenotype is conserved in *Caenorhabditis elegans *and that the underlying mechanism is based on a redirection of the metabolic flux from glycolysis to the pentose phosphate pathway, altering the redox equilibrium of the cytoplasmic NADP(H) pool. Remarkably, another key glycolytic enzyme, glyceraldehyde-3-phosphate dehydrogenase (GAPDH), is known to be inactivated in response to various oxidant treatments, and we show that this provokes a similar redirection of the metabolic flux.

**Conclusion:**

The naturally occurring inactivation of GAPDH functions as a metabolic switch for rerouting the carbohydrate flux to counteract oxidative stress. As a consequence, altering the homoeostasis of cytoplasmic metabolites is a fundamental mechanism for balancing the redox state of eukaryotic cells under stress conditions.

## Background

Reactive oxygen species (ROS) cause damage to cellular processes in all living organisms and contribute to a number of human disorders such as cancer, cardiovascular diseases, stroke, and late-onset neurodegenerative disorders, and to the aging process itself. To cope with the fatal cellular consequences triggered by ROS, eukaryotic cells have evolved a number of defense and repair mechanisms, which are based on enzymatic as well as non-enzymatic processes and appear to be highly conserved from unicellular to multicellular eukaryotes. In bacteria and yeast, these antioxidant defense mechanisms are partially induced on the basis of changes in global gene expression [1,2]. However, a recent study analyzing a number of genetic and environmental perturbations in *Escherichia coli *demonstrated that the changes in the transcriptome and proteome are unexpectedly small [3]. Moreover, the transcription of genes encoding enzymes capable of neutralizing ROS is not generally increased in mammalian cells that are subjected to oxidative stress [4].

In all organisms studied, however, treatment with oxidants prompts immediate *de novo *post-translational modifications of a number of proteins, probably affecting their localization and functionality. One of the key targets of those processes is the glycolytic enzyme glyceraldehyde-3-phosphate dehydrogenase (GAPDH), which catalyzes the reversible oxidative phosphorylation of glyceraldehyde-3-phosphate (gly3p) to 1,3-bisphosphoglycerate. Remarkably, in response to various oxidant treatments this enzyme is inactivated and transported into the nucleus of the cell, and has been found S-nitrosylated, S-thiolated, S-glutathionylated, carbonylated and ADP-ribosylated in numerous cell types and organisms under these conditions [5-10]. Recently, we discovered that yeast cells with reduced catalytic activity of another key glycolytic enzyme, triose-phosphate isomerase (TPI), are highly resistant to the oxidant diamide [11]. This essential enzyme precedes GAPDH in glycolysis, catalyzing the interconversion of dihydroxyacetone phosphate (dhap) and gly3p, the substrate of GAPDH, and a reduction in its activity results in an elevated cellular dhap concentration [12-14]. In this light, it is remarkable that the expression of a subset of glycolytic proteins and proteins implicated in related pathways is repressed, while the expression of a few enzymes involved in the pentose phosphate pathway (PPP), which is directly connected to the glycolytic pathway, is induced under oxidative stress conditions [1]. Furthermore, enhanced activity of the PPP has been observed in neonatal rat cardiomyocytes and in human epithelial cells under oxidative stress conditions [15,16]. Enzymes of the PPP are crucial for maintaining the cytoplasmic NADPH concentration, which provides the redox power for known antioxidant systems [17,18]. The observations above suggest that alterations in the carbohydrate metabolism could be central for cellular protection against ROS and, moreover, that cells reroute the carbohydrate flux from glycolysis to the PPP to counteract perturbations in the cytoplasmic redox state. However, direct evidence for this hypothesis is missing so far. By combining genetic and quantitative metabolite analyses along with *in silico *modeling, we present the first direct proof that eukaryotic cells indeed actively reroute the metabolic flux from glycolysis to the PPP as an immediate and protective response to counteract oxidative stress.

## Results

### Reduced intracellular TPI concentration results in enhanced oxidant resistance of *Saccharomyces cerevisiae *and *Caenorhabditis elegans*

**Figure 1:**
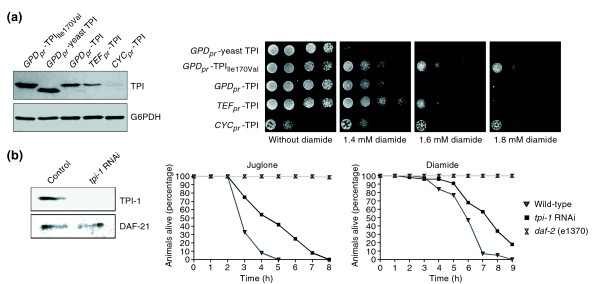
Reduced triosephosphate isomerase (TPI) activity increases oxidant resistance of *S. cerevisiae *and *C. elegans*. **(a) **The left panel shows a Western blot analysis of yeast cells expressing wild-type human TPI under the control of promoters of different strengths: *GPD1 *(*GPD*_*pr*_), *TEF1 *(*TEF*_*pr*_), and *CYC1 *(*CYC*_*pr*_). Yeast cells expressing human TPI_Ile170Val _or yeast TPI under the control of the strong *GPD1 *promoter were used as controls. Equal loading of the lysates was controlled by visualizing G6PDH. The right panel shows yeast cells expressing yeast TPI and human TPIIle170Val controlled by the *GPD1 *promoter or yeast expressing wild-type human TPI controlled by the *GPD1*, *TEF1 *or *CYC1 *promoters, respectively. Yeast were spotted as fivefold serial dilutions on SC medium supplemented with different concentrations of diamide. Plates were incubated at 30°C for 3 days. **(b) **The left panel shows western blot analysis of cell extracts prepared from adult *C. elegans *that were fed with *E. coli *producing double-stranded RNA of the *C. elegans tpi-1 *gene (Y17G7B.7) (*tpi-1 *RNAi) or harboring the empty plasmid L4440 (control). The right panel shows the effects of the oxidants juglone and diamide on these worms. After feeding with *E. coli *as described above, worms were placed on agar plates supplemented with juglone or diamide. Multi-resistant *daf-2 *(e1370) mutant worms were included in every experiment as controls.

We reported earlier that a change of the amino acid isoleucine to valine at position 170 in the human TPI protein (TPI_Ile170Val_) causes a reduction of about 70% in the enzyme's catalytic activity [11]. Interestingly, we discovered that yeast cells expressing this human TPI variant exhibit increased resistance to the oxidant diamide (N,N,N',N'-tetramethylazodicarboxamide, Chemical Abstracts Service (CAS) No. 10465-78-8) compared with isogenic yeast cells expressing wild-type human TPI, indicating that low TPI activity confers resistance to specific conditions of oxidative stress. The synthetic reagent diamide is known to oxidize cellular thiols, especially protein-integrated cysteines [19], provoking a rapid decrease in cellular glutathione and hence causing oxidative stress. To dissect the underlying mechanism, we first analyzed whether decreasing the expression level of wild-type human TPI would result in a similar phenotype. For this, we generated plasmids for the expression of wild-type human TPI under the control of established yeast promoters of different strengths, namely the *CYC1*, *TEF1 *and *GPD1 *promoters [20]. Subsequently, the Δ*tpi1 *strain MR100, which is deleted for the yeast *TPI1 *gene and is inviable on medium containing glucose as sole carbon source, was transformed with the respective plasmids along with control plasmids encoding yeast TPI_Ile170Val _or yeast TPI. Single colonies were selected and the intracellular TPI concentration of plate-grown yeast cells was analyzed (Figure 1a, left panel). As expected, yeast cells expressing the different TPI proteins under the strong *GPD1 *promoter had a higher TPI concentration compared with cells in which the expression was controlled by the intermediate *TEF1 *or the weak *CYC1 *promoter. Next, we spotted the respective yeast cells onto medium supplemented with differing diamide concentrations. As shown in Figure 1a (right panel), yeast cells expressing human TPI under the control of the weakest promoter used, the *CYC1 *promoter, grew slowly on standard medium compared with the other yeast strains. Notably, growth of these cells on plates containing 1.6–1.8 mM diamide was comparable to the growth of control yeast cells expressing the TPI_Ile170Val _protein with reduced catalytic activity, demonstrating that a reduction in TPI expression or specific activity confers resistance against this oxidant. Furthermore, yeast cells expressing wild-type human TPI under the control of the intermediate *TEF1 *promoter grew on medium containing 1.8 mM diamide, albeit to a much lesser extent than yeast cells in which TPI expression is controlled by the weak *CYC1 *promoter. This finding excludes the possibility that the observed oxidant resistance of yeast cells with *CYC1*-controlled TPI expression is based solely on their slower growth rate. In support of this, yeast cells in which the strong *GPD1 *promoter controls TPI expression did not grow at all on medium containing 1.6–1.8 mM diamide. Moreover, yeast cells ectopically expressing yeast TPI from the same promoter, which is approximately 30% more active than human wild-type TPI in yeast [11], were even more sensitive to diamide. Thus, diminishing the expression level or activity of TPI increases the diamide tolerance of yeast.

Next, we investigated whether this phenomenon is conserved in multicellular eukaryotes, and addressed this by using *Caenorhabditis elegans *as a model. RNA interference (RNAi) technology was used to reduce (knock down) the intracellular concentration of TPI by feeding worms with *E. coli *producing double-stranded RNA of the *C. elegans tpi-1 *gene (Y17G7B.7); the empty RNAi vector (L4440) was used as control. The reduction of the intracellular TPI concentration was analyzed by immunoblotting (Figure 1b, left panel). Then, *tpi-1 *knock-down worms were placed on agar plates supplemented with the oxidant juglone (5-hydroxy-1,4-naphthalenedione, CAS No. 481-39-0), a natural naphthoquinone found particularly in the black walnut *Juglans nigra*. This oxidant triggers the generation of superoxide radicals as a result of its capacity for redox cycling that involves a one-electron redox reaction generating semiquinone and superoxide radicals [21]. As controls, multi-stress-resistant *daf-2 *mutant worms were included in every experiment, and surviving worms were counted each hour. Worms with reduced TPI concentration placed on 10 μM juglone plates survived significantly longer than wild-type animals under the same conditions (Figure 1b, middle panel). In addition, the average survival time of wild-type worms on 10 μM juglone plates was 4.2 ± 0.8 hours, whereas TPI knock-down animals survived for 5.5 ± 0.4 hours (*p *value of 1.13e^-07^, see Additional data file 1 for more quantitative information). We also carried out the same set of experiments using the oxidant diamide, which is not usually used in *C. elegans *laboratories. We discovered that worms were highly resistant to this oxidant, and very high concentrations had to be applied for growth inhibition (data not shown). Notably, we showed, by applying as much as 250 mM diamide, that TPI knock-down worms displayed an increased resistance (Figure 1b, right panel). The knock-down of TPI resulted in a greater average survival time compared with wild-type animals (8.6 ± 0.3 hours vs 7.5 ± 0.3 hours, *p *value of 0.011, see Additional data file 1). Thus, these experiments clearly show that a reduction in TPI activity increases oxidant resistance of the multicellular eukaryote *C. elegans*.

### Reduced TPI activity protects against diamide by increasing the activity of the PPP

**Figure 2:**
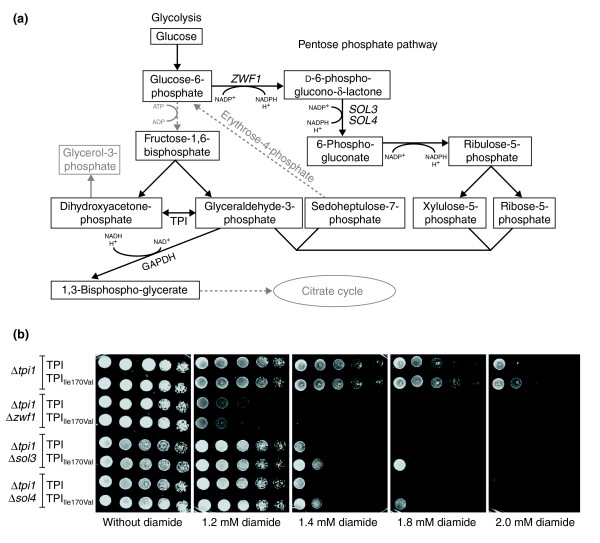
Reduced TPI activity protects against diamide by increasing the metabolic flux through the PPP. **(a) **Schematic illustration of a subset of biochemical reactions of the glycolytic pathway (left) and the associated pentose phosphate pathway (right). Solid lines represent direct, one-step biochemical reactions, and indirect, multi-step reactions are represented as dotted lines. GAPDH, glyceraldehyde-3-phosphate dehydrogenase. **(b) **Yeast deletion strains Δ*tpi1*Δ*zwf1*, Δ*tpi1*Δ*sol3*, and Δ*tpi1*Δ*sol4 *expressing wild-type human TPI or TPI_Ile170Val _were spotted as fivefold serial dilutions on synthetic media supplemented with different concentrations of diamide, and plates were incubated at 30°C.

We next aimed to dissect the molecular basis for the observed diamide resistance in yeast by genetic means. The glycolytic pathway is directly interconnected with the PPP, which is one of the key pathways in reducing the pyridine nucleotide NADP^+ ^to NADPH within the eukaryotic cytoplasm and, hence, one of the main cellular sources of the cytoplasmic NADPH that is required as a redox cofactor by the main antioxidant enzymes to neutralize ROS (see [18] for a review). We speculated that the inactivation of TPI, resulting in a block on glycolysis, should counteract oxidative stress by elevating the metabolic flux of the PPP (Figure 2a). To test this assumption, we aimed to genetically target the first two steps of the PPP. As indicated in Figure 2a, the rate-limiting generation of D-6-phosphoglucono-δ-lactone from glucose-6-phosphate (g6p), the metabolite for which glycolysis and PPP are competing for, is catalyzed by the yeast glucose-6-phosphate dehydrogenase (G6PDH) Zwf1p [17,22]. In the second step of the PPP, this metabolite is converted by the paralogous 6-phospho-gluconolactonases Sol3p and Sol4p into 6-phosphogluconate [23]. Blocking these two essential steps would impair the activity of the PPP and lessen the observed protective effect of reduced TPI activity.

We therefore generated yeast strains expressing wild-type human TPI or TPI_Ile170Val _in which the yeast genes *TPI1 *and *ZWF1*, *TPI1 *and *SOL3*, or *TPI1 *and *SOL4 *were deleted. These strains were then spotted as fivefold serial dilutions on synthetic media containing different concentrations of diamide. As shown in Figure 2b, growth of the corresponding Δ*tpi1*Δ*zwf1*, Δ*tpi1*Δ*sol3 *and Δ*tpi1*Δ*sol4 *yeast cells was strongly reduced compared with the respective Δ*tpi1 *yeast cells on medium containing 1.4–2.0 mM diamide. Notably, Δ*tpi1*Δ*zwf1 *cells, which are unable to metabolize g6p to enter the PPP, exhibited the strongest sensitivity; these cells already grew poorly on medium supplemented with 1.2 mM diamide. As expected, Δ*tpi1 *yeast cells expressing TPI_Ile170Val _grew better on media containing high diamide concentrations compared with yeast cells expressing wild-type TPI, confirming the protective effect observed earlier. Strikingly, the protective effect of TPI_Ile170Val _was no longer observed in Δ*tpi1*Δ*zwf1 *cells, in which the interplay between glycolysis and the PPP is blocked. In addition, the protective effect of TPI_Ile170Val _against diamide in Δ*tpi1*Δ*sol3 *and Δ*tpi1*Δ*sol4 *cells was detectable, but weaker. This was expected, since Δ*tpi1*Δ*sol3 *and Δ*tpi1*Δ*sol4 *cells are still able to convert D-6-phosphoglucono-δ-lactone to 6-phosphogluconate by reducing one equivalent of NADP^+^ due to the presence of one wild-type copy of either *SOL4 *or *SOL3*. Thus, these experiments clearly demonstrate that the protective effect of reduced TPI activity is indeed based on the activity of the PPP and is absent if the first and rate-limiting step of the PPP is inhibited.

### Preventing the accumulation of NADPH sensitizes yeast cells to diamide

**Figure 3:**
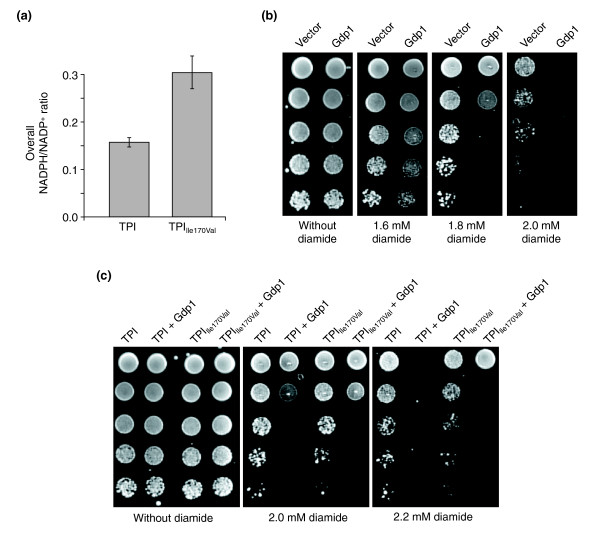
Reduced TPI activity protects against diamide by increasing NADPH. **(a) ***S. cerevisiae *strains MR101 and MR105 were grown in duplicate to mid-log phase, pyridine nucleotides were extracted, and LC-MS/MS measurements were performed in triplicate. MR105 cells expressing TPI_Ile170Val _had a higher overall NADPH/NADP^+ ^ratio compared with MR101 cells expressing wild-type TPI. **(b) ***S. cerevisiae *strain BY4741 was transformed with an empty 2μ plasmid or with a 2μ plasmid encoding *K. lactis *GDP1 (p1696). Afterwards, single transformants were selected, grown overnight and the same number of cells were spotted as fivefold serial dilutions on agar plates supplemented with different concentrations of diamide. Growth was monitored after plates were incubated at 30°C for 3 days. **(c) **The isogenic yeast strains MR101 expressing wild-type human TPI or MR105 expressing human TPI_Ile170Val _were transformed with plasmids for expression of *K. lactis GDP1 *and processed as described in (b).

As most antioxidant enzymes are coupled to NADPH as a redox cofactor and a functional defense mechanism against oxidative stress depends upon the availability of NADPH, we hypothesized that increased activity of the PPP might protect against oxidative stress due to the enhanced cellular production of this molecule. To test this hypothesis, we set out to measure the overall NADPH/NADP^+ ^ratio of MR101 cells expressing human wild-type TPI and MR105 cells expressing TPI_Ile170Val_. The respective strains were grown in duplicate to mid-log phase and pyridine nucleotides were extracted simultaneously as described by Noack *et al*. [24], performing a three-step protocol that is based on a 34:24:1 phenol:chloroform:isoamyl-alcohol pyridine-nucleotide extraction that is followed by two diethylether re-extractions of the aqueous phase. As measured by liquid chromatography - tandem mass spectrometry (LC-MS/MS), the overall NADPH/NADP^+ ^ratio was indeed highly increased in MR105 cells expressing TPI_Ile170Val _in comparison to MR101 cells expressing wild-type TPI (Figure 3a). Although the LC-MS/MS analysis does not allow discrimination between cytoplasmic and mitochondrial NADP(H), the measurements clearly show that the redox equilibrium of the NADP(H) pool strongly shifts towards NADPH in cells with reduced TPI activity; the increase in the sole cytoplasmic NADPH/NADP^+ ^ratio is expected to be even higher than the measured values of the overall NADPH/NADP^+ ^ratio.

To substantiate these results *in vivo *and to correlate with the observed oxidant-resistance phenotype, we investigated the effect of the Gdp1 protein of the yeast *Kluyveromyces lactis*, a phosphorylating (NADP^+^-dependent) glyceraldehyde-3-phosphate dehydrogenase (GenBank accession number CAD23142, Enzyme Commission classification EC 1.2.1.13 [25]). Except for *K. lactis*, this enzyme has not been detected in non-plant eukaryotes; it was discovered in a screen designed to find suppressors for the lethal effects of phosphoglucose isomerase (Pgi1) deletion in *S. cerevisiae *on glucose media [25]. The absence of Pgi1p is lethal for *S. cerevisiae *on standard media, because a strong NADPH accumulation occurs at the expense of its oxidized form [26].

Expression of *K. lactis *Gdp1 rescued the lethality of Δ*pgi1 S. cerevisiae *cells because it catalyzes the oxidation of NADPH to NADP^+ ^[25], thus preventing the accumulation of NADPH in Δ*pgi1 *cells [25]. Gdp1 can therefore be applied *in vivo *to analyze the impact of NADPH accumulation in regard to the observed oxidant resistance of yeast cells with reduced TPI activity. To do this, we transformed the yeast strain BY4741 with either a plasmid encoding *K. lactis GDP1 *under the control of a constitutive promoter or with an empty control plasmid and selected the respective transformants on plates of synthetic complete (SC) medium lacking uracil (SC^-ura^). Yeast cultures were then grown and spotted as fivefold dilution series on solid medium supplemented with varying concentrations of diamide. As shown in Figure 3b, yeast cells expressing Gdp1 were highly sensitive to diamide in the concentration range of 1.8–2.0 mM compared with control cells, indicating that the cellular NADPH/NADP^+ ^balance is crucial for the cellular resistance to diamide. To further validate that increased activity of the PPP leading to an elevated cellular reduction of NADP^+ ^to NADPH underlies the observed resistance to diamide, we addressed the impact of *GDP1 *expression in Δ*tpi1 *yeast strains expressing the human protein TPI_Ile170Val_. We observed that growth of Δ*tpi1 *yeast expressing the human TPI proteins and *K. lactis *Gdp1 was strongly impaired on medium supplemented with 2.0 or 2.2 mM diamide (Figure 3c). Remarkably, the effects of *GDP1 *expression were less dramatic in yeast cells expressing TPI_Ile170Val_, which have an increased NADPH/NADP^+ ^ratio. Thus, these results suggest that the enhanced diamide resistance of yeast cells with reduced TPI activity is based on increased conversion of NADP^+ ^to NADPH within the PPP.

### Inactivation of TPI and GAPDH increases the concentration of PPP metabolites

We observed in an earlier study [11] that yeast cells with reduced TPI activity are not resistant to oxidative stress caused by hydroperoxides such as hydrogen peroxide (H_2_O_2_), cumene hydroperoxide or tert-butylhydroperoxide. Strikingly, treatment of yeast cells with these oxidants leads to a rapid inactivation of GAPDH; however, this inactivation is not observed when cells are treated with diamide [6,27]. As GAPDH is the first enzyme downstream of TPI, we speculated that the block of GAPDH activity in hydroperoxide-treated yeast cells prevents the protective effects of reduced TPI activity. This hypothesis would imply that cells do inactivate GAPDH to reroute the metabolic flux to the PPP for protection against ROS. To corroborate this, we comprehensively measured a number of glycolytic and PPP metabolites, and compared changes between their intracellular concentration in yeast cells expressing TPI variants with reduced activity and wild-type yeast cells treated with H_2_O_2_. For this analysis, the corresponding yeast cultures were grown in rich medium (YPD) to an equal optical density and lysed as described in Materials and methods. In the quantitative metabolomic analyses, we focused on the metabolites dhap, glucose-6-phosphate/fructose-6-phosphate (g6p), 6-phosphogluconate (6pg), ribose-5-phosphate (r5p), xylulose-5-phosphate/ribulose-5-phosphate (x5p), sedoheptulose-7-phosphate (s7p), glyceraldehyde-3-phosphate (gly3p) and glycerol-3-phosphate (gol3p). Quantification was carried out using LC-MS/MS.

**Figure 4:**
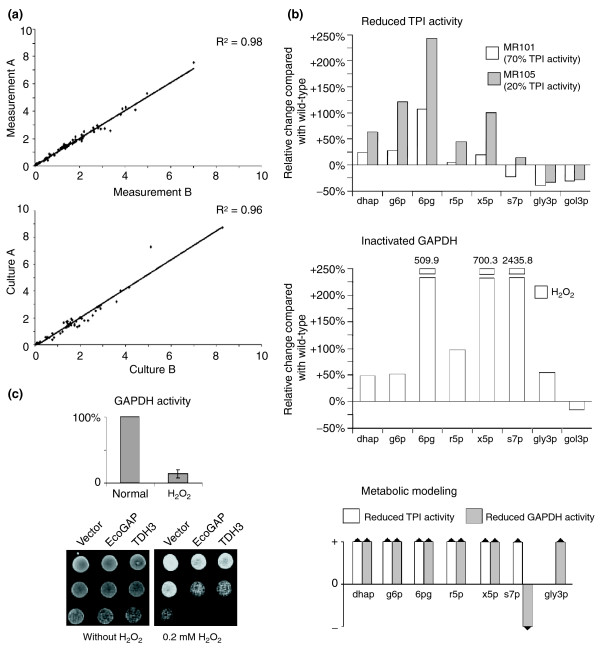
TPI and GAPDH inactivation increases the concentration of PPP metabolites. **(a) **For quality control of the metabolite quantifications and for analyzing the technical reproducibility, each metabolite was measured in duplicate (top panel). For analyzing the biological reproducibility, the metabolite concentrations were measured from cultures grown in parallel (bottom panel). Please note that for the purpose of illustration values greater than 10 are not shown. The complete plots are presented in Additional data file 3. **(b) **Upper panel, changes in metabolite levels in yeast strains with differing TPI activity. Lysates of yeast strains BY4741 (100% TPI activity), MR101 (70% TPI activity) and MR105 (20% TPI activity) were prepared and metabolites were quantified by LC-MS/MS. The absolute metabolite concentrations of MR101 and MR105 yeast were normalized and plotted as change given in percent relative to the wild-type (BY4741) strain. Middle panel, changes in metabolite levels in yeast with GAPDH inactivation. Cultures of strain BY4741 were treated with H_2_O_2 _or left untreated. The relative changes of the various metabolites of the H_2_O_2_-treated cells in comparison to untreated cells were plotted. Bottom panel, predicted qualitative changes in metabolite concentrations using the non-fitted metabolic model. Note that for technical reasons, the abbreviation g6p refers to the sum of glucose-6-phosphate and fructose-6-phosphate and x5p to the sum of xylulose-5-phosphate and ribulose-5-phosphate. **(c) **Upper panel, GAPDH activity in yeast cells treated with and without H_2_O_2 _as in (b). Lower panel, effect of H_2_O_2_ on wild-type yeast cells transformed with the 2μ plasmids p423GPD, p423GPD-*EcoGAP *encoding *E. coli *GAPDH, or p423GPD-*TDH3 *encoding the yeast GAPDH Tdh3p. Transformants were selected, grown overnight and the same number of cells were spotted as fivefold serial dilutions on SC^-his-ade ^media supplemented with H_2_O_2 _as indicated.

We first set out to analyze the experimental quality of our measurements, and prepared two samples from each culture for measurements of the various metabolites. The measurements of the parallel samples were plotted on a two-dimensional graph and analyzed statistically (Figure 4a, upper panel). The coefficient of determination (*R*^2^) equaled 0.9989 when including all measurements (0.98 for values smaller than 10), indicating high reproducibility of the analysis. Next, we assayed the comparability of the metabolite content of yeast cultures cultivated in duplicate. Two lysate samples of each culture were prepared in parallel and the metabolite content of each sample was measured in duplicate. The average concentration of each metabolite was plotted on a two-dimensional graph and analyzed statistically (Figure 4a, lower panel). Here, the *R*^2 ^value of 0.995 (0.96 analyzing values smaller than 10) demonstrated excellent comparability of the metabolite content of yeast cultures grown in parallel.

Finally, we calculated the relative alterations in the cellular metabolite concentrations of two different yeast strains – MR101, which expresses human TPI, and MR105, which expresses human TPI_Ile170Val _– compared with the isogenic wild-type strain BY4741 (Figure 4b, upper panel). MR101 yeast exhibits 70% and MR105 20% overall TPI activity compared with the wild-type strain BY4741, as determined by the TPI activity assay described earlier [11]. As expected, we detected increased levels of the TPI substrate dhap in yeast cells with reduced TPI activity, as previously observed in human cell extracts and in yeast [13,14]. The moderately reduced TPI activity in MR101 cells caused an increase in the intracellular dhap concentration of 24.9% compared with the level of wild-type strain BY4741. A strong increase in dhap concentration was measured in lysates prepared from MR105 cells and we also found that the concentration of g6p was increased in MR101 and MR105 cells. As mentioned previously, g6p is converted by glycolysis and the PPP and is rate-limiting for their activity (Figure 2a). In addition, the intracellular concentration of the metabolites 6pg, r5p and x5p, all generated in the PPP, were elevated in MR101 and MR105 cells. Notably, the concentration changes of these metabolites followed the trend in TPI activity in both these strains: the lower the TPI activity, the higher the increase in metabolite concentration. As expected, the cellular concentration of the TPI product, gly3p, was decreased in both strains. Furthermore, the metabolite s7p was decreased in MR101 cells, but increased in MR105 cells. This unexpected finding could potentially reflect a change in the equilibrium between gly3p and s7p, as both metabolites are simultaneously required by the yeast transketolases Tkl1p and Tkl2p; however, an adequate explanation cannot be given at present. Thus, these experiments clearly show that a decrease in cellular TPI activity results in elevated levels of almost all the metabolites of the PPP.

We next analyzed whether treatment of yeast cells with H_2_O_2_, known to cause inactivation of GAPDH [10,27,28], would result in a similar rerouting of the carbohydrate flux. Wild-type cells were treated with H_2_O_2 _for 30 minutes as described [28], collected by centrifugation, and the GAPDH activity was measured as described in Materials and methods. As shown in Figure 4c (upper panel), GAPDH was inactivated in H_2_O_2_-treated yeast cells. To further demonstrate the contribution of GAPDH to resistance to H_2_O_2_, we investigated the H_2_O_2_-tolerance of yeast cells overexpressing either the most abundant yeast GAPDH paralog, Tdh3p, or the *E. coli *GAPDH, EcoGAP. As anticipated, cells overexpressing Tdh3p or EcoGAP were more sensitive to H_2_O_2 _treatment compared with cells harboring the empty vector (Figure 4c, lower panel). Moreover, Tdh3p or EcoGAP overexpression in another yeast background, the W303 derivate Y2546, also caused sensitivity to H_2_O_2 _(data not shown). Subsequently, we analyzed the changes in metabolite concentrations of H_2_O_2_-treated yeast cells and found that concentrations of all measured PPP metabolites were greatly increased (Figure 4b, middle panel). The greatest increases were observed for 6pg, x5p and s7p. Moreover, we found decreased concentrations of the glycolytic metabolite gol3p, which is generated intracellularly from dhap by the enzyme Gpd1p (also known as Hor1p). Strikingly, all measured metabolites showed a similar tendency in the case of inactivated GADPH as was observed for low TPI activity, with the exception of gly3p. Indeed, gly3p represents the metabolic intermediate of both enzymes. These results show that yeast cells reroute the carbohydrate flux in response to H_2_O_2 _treatment in the same manner as cells with low TPI activity. This implies that rerouting of the metabolic flux is a basic mechanism in counteracting oxidative stress that is naturally switched on in the course of GAPDH inactivation.

### Mathematical modeling and computer simulations

Because our experimental data imply that inactivation of GAPDH may serve as a cellular switch to reroute the metabolic flux from glycolysis to the PPP under oxidative stress conditions, we set out to develop a mathematical model that describes the dynamic behavior of the metabolic reactions under consideration. Most of the reactions involved have been intensely studied *in vitro* and, hence, sufficient kinetic parameters (K_m_, V_max_) are available for modeling and simulating the entire pathway *in silico*. For this, we modeled enzymatic reactions (see Additional data file 2) as a set of ordinary differential equations using the CellDesigner software [29]. The model allows calculation of the concentrations of 19 different metabolites, the amount of high-energy phosphate groups (P), and the NAD^+^/NADH and NADP^+^/NADPH ratios. Three types of *in silico *simulations were run: with normal TPI and GAPDH activity; with 25% residual TPI activity; and with 25% residual GAPDH activity. The results of these simulations were compared with the LC-MS/MS measurements from wild-type yeast, from strain MR105, which expresses TPI_Ile170Val_, and from H_2_O_2_-treated wild-type cells with inactivated GAPDH. The simulations revealed that 13 of the 14 qualitative changes in metabolite concentrations were correctly predicted by the mathematical model (Figure 4b, lower panel). A difference between the experimental data and the predictions was only observed for the metabolite s7p. The simulations predicted a decline of s7p in H_2_O_2_-treated yeast cells whereas the respective experiments showed that the concentration of s7p increased.

**Figure 5:**
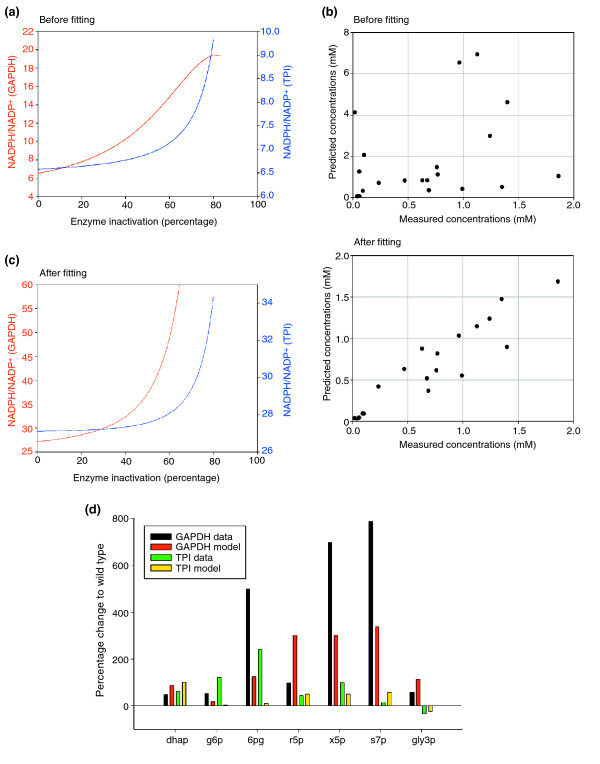
*In silico *model for the interplay of glycolysis and the pentose phosphate pathway in response to GAPDH or TPI inactivation. **(a) **Predicted changes of the cytoplasmic NADPH/NADP^+ ^ratio of the unfitted model. The NADPH/NADP^+ ^ratio increases in correlation with the rate of TPI (blue) or GAPDH (red) inactivation. **(b) **Quantitative accuracy of the metabolic model before and after parameter fitting. Upper panel, 21 measured metabolite concentrations (seven metabolites under three conditions) are plotted against the predicted values before fitting. Lower panel, data versus prediction after parameter fitting. **(c) **As (a), but after parameter fitting. **(d) **Comparison of quantitative predictions made with the parameter-fitted and the measured metabolite concentrations. Changes relative to the wild-type strain values are shown. Black and red bars correspond to yeast cells with inactivated GAPDH, green and yellow bars correspond to reduced TPI activity.

As the qualitative predictions of the unfitted model matched well with the experimental data set, we calculated the influence of reduced TPI or GAPDH activity on the cellular NADPH/NADP^+ ^ratio without any further parameter fitting. Like other mathematical models [30,31], our model is based on the fact that the nicotinamide nucleotide moiety is conserved: that is, that the sum of cellular NADP^+ ^and NADPH is constant. The free-energy change (Δ*G*) of a reaction is given by Δ*G* = Δ*G^0'^*+RT⋅ln(k), (where Δ*G*^0'^is the standard free-energy change and *k *is the equilibrium constant). For a redox reaction involving NADPH and NADP^+ ^(*k *= reduced form/oxidized form), it is therefore the NADPH/NADP^+ ^ratio, and not the absolute concentrations, that drives the reaction. Hence, we calculated the corresponding steady-state values of the NADPH/NADP^+ ^ratio depending on the activity of GAPDH or TPI using the program Copasi 4B20 [32] (Figure 5a). Reduction of TPI activity resulted in an increased NADPH/NADP^+ ^ratio from approximately 6.5 to 9. The simulated reduction in GAPDH activity resulted in an even greater increase in the NADPH/NADP^+ ^ratio, from approximately 6.5 to 19. Taken together, simulations using a dynamic, unfitted mathematical model corroborate the experimental finding that reduced TPI and GAPDH activities redirect the carbohydrate flux. Moreover, the model predicts an elevated NADPH/NADP^+ ^ratio if the activity of the PPP is increased, a result that agrees with earlier experimental observations.

Although the qualitative results of the simulations fit very well with the measurements without modifying any of the kinetic parameters taken from the literature, it should be noted that the kinetic constants were determined using enzymes from five different species (human, cow, rabbit, yeast, *E. coli*) in different laboratories over a period of more than three decades. Consequently, it cannot be expected that the simulations coincide quantitatively with the measured metabolite concentrations. However, the high-quality LC-MS/MS data allowed us to adjust the numerical values of the kinetic parameters so that the predicted metabolite concentrations agree better with the measured ones. For this parameter fitting, we used the measured metabolite concentrations (see Figure 4 and Additional data files 2 and 3) and the Copasi software using the Hook and Jeeves algorithm with the constraint that the literature parameters can only vary by a factor of 4. The results of this fitting process are shown in summary form in Figure 5b. The 21 measured concentrations (seven metabolites, three conditions) were plotted against the concentrations predicted by the model. Before fitting (Figure 5b, upper panel), a general trend existed that large predicted concentrations corresponded to large measured concentrations, but the correlation was not sufficient for quantitative prediction. After fitting, however, the experimental data and the mathematical model showed a much better correlation (Figure 5b, lower panel). Here, one of the data points deserves special attention: the measured s7p concentration in H_2_O_2_-treated yeast cells was extremely high (17 mM, see Additional data file 3 for metabolite concentrations), which differs greatly from the predicted value of 0.5 mM before fitting and 2.27 mM after fitting. The most likely explanation is that s7p undergoes reactions that were not included in the model or that have not been identified. For instance, the enzyme heptulokinase is known to phosphorylate sedoheptulose to sedoheptulose-7-phosphate, and vice versa, but a dependence of this reaction on GAPDH has not been reported so far. Moreover, H_2_O_2 _treatment could influence other parts of the reaction network, and indeed, transketolase activity appears to be reduced in oxidant-treated cells [33,34]. Nonetheless, the oxidant sensitivity of the transketolase is not sufficient to explain the unexpected concentration changes of s7p observed in yeast cells with reduced TPI activity as described above. Thus, the most likely explanation for the phenomenon remains that s7p is involved in as yet unknown cellular processes that are implicated in the oxidative stress response.

To improve the visualization of the quantitative output of our calculations, we grouped the quantitative results of the experimental measurements and the calculations from the fitted model by each metabolite (Figure 5d). Because the fitted model showed improved correlation between the experimental data and the mathematical model, we reanalyzed the prediction made for the intracellular NADPH/NADP^+ ^ratio (Figure 5a) with the quantitatively fitted model. The simulation confirmed the earlier result that inactivating TPI or GAPDH leads to increased NADPH/NADP^+ ^ratios (see Figures 5c and 3a). Inactivation of GAPDH again resulted in a greater increase in the cellular NADPH/NADP^+ ^ratio than that resulting from reduced TPI activity. Thus, GAPDH can be regarded as a cellular switch causing rerouting between both metabolic pathways, and the naturally-occurring GAPDH inactivation appears to be more effective and sufficient in terms of redirecting the carbohydrate flux from glycolysis to the PPP. Notably, our modeling approach revealed that the experimentally observed alteration in s7p levels cannot be explained by the current knowledge of the kinetics of glycolysis and PPP. It would be therefore of interest to focus in future on the sedoheptulose metabolism in order to close this gap. The good quantitative agreement between the model and the experimental results underlines the solidness of the model and provides a firm base for further comprehensive simulations of eukaryotic carbohydrate metabolism integrating other metabolic pathways that are associated with glycolysis and the PPP.

### PPP activity is a regulator of normal lifespan of *S. cerevisiae *and *C. elegans*

**Figure 6:**
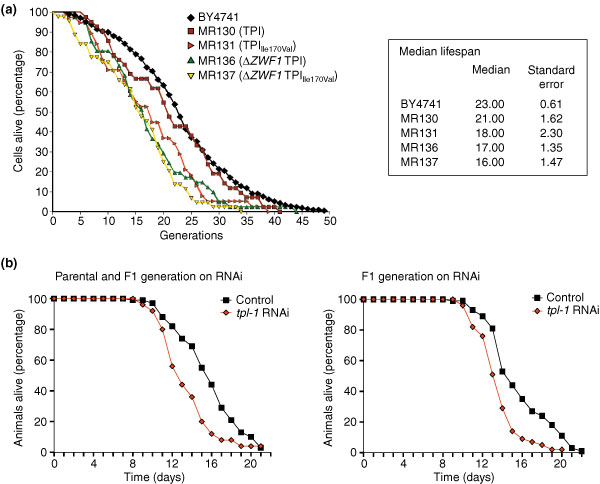
Lifespan analysis of *S. cerevisiae *and *C. elegans*. **(a) **The median replicative lifespan of yeast strains BY4741, MR130, MR131, MR136, and MR137 was determined by counting surviving mother cells per generation. **(b) **For the lifespan analysis of *C. elegans *the parental and the F1 generation (left panel, 117 wild-type and 80 *tpi-1 *RNAi animals were analyzed) or the F1 generation only (right panel, 74 wild-type and 47 *tpi-1 *RNAi animals) were placed on the respective agar plates and survival of the worms was monitored every day.

Much evidence exists that oxidative damage to diverse cellular components is implicated in the aging process. Intriguingly, several genetic mutations that have been reported to increase the overall lifespan of a variety of organisms lead to increased oxidant-resistance as well [35]. However, concluding the converse, that genetic mutations mediating oxidant resistance generally increase the overall or maximum lifespan, is not feasible. Cultured foreskin fibroblasts lacking the human ortholog of *ZWF1*, hG6PDH, display premature aging [36], and so we set out to analyze whether rerouting the carbohydrate flux influences the aging process. We first determined the median replicative lifespan of Δ*tpi1 *and Δ*tpi1*Δ*zwf1 *yeast cells expressing TPI or TPI_Ile170Val_. As shown in Figure 6a, MR130 yeast cells expressing wild-type TPI had a median replicative lifespan of 21 cell divisions, a number that did not differ significantly from the lifespan of the parent strain BY4741. However, isogenic Δ*zwf1 *yeast cells, which are not capable of redirecting the carbohydrate flux from glycolysis to the PPP, had a statistically significant lower replicative lifespan of 17 cell divisions. Moreover, MR131 yeast expressing TPI_Ile170Val_, which had an average of 18 cell divisions, did not significantly differ in their median replicative lifespan from Δ*tpi1*Δ*zwf1 *cells expressing wild-type TPI (MR136), but were short-lived compared with the respective wild-type cells, which had a median lifespan of 21 cell divisions. Finally, the median lifespan of Δ*tpi1*Δ*zwf1 *cells expressing TPI_Ile170Val _(MR137) was even lower, at only 16 cell divisions. These results show that proper interplay between glycolysis and the PPP is required for normal lifespan in yeast. Interestingly, we observed additive effects of reduced TPI activity and *ZWF1 *deletion on the replicative lifespan, indicating that the negative influence of reduced TPI activity on replicative aging does not depend on the activity of the PPP.

We also analyzed the effect of reduced TPI activity on the lifespan of *C. elegans*. Like the stress experiments, the lifespan experiments were carried out with worms that were fed *E. coli *producing double-stranded RNA for knockdown of the *C. elegans tpi-1 *gene (Y17G7B.7) with empty RNAi vector as control. Surviving animals were counted daily. We observed that the mean lifespan of wild-type worms on the vector control was 15.7 ± 0.9 days, whereas the average lifespan of *tpi-1 *knock-down worms, with 14.4 ± 0.9 days, was significantly shorter (p = 0.0016; Figure 6b, right panel). Also, the maximum lifespan of 21.0 ± 1.4 days for *tpi-1 *knock-down worms was shorter than the 23.0 ± 0 day lifespan of wild-type worms (see Additional data file 1 for more details). Furthermore, a similar shortening of lifespan was observed when TPI expression was also reduced, beginning with late larval stage L4 parental generation and during the development of the F1 (Figure 6b, left panel). These findings confirm earlier studies showing that normal activity of the PPP is central to the native lifespan of eukaryotic organisms. In addition, our results revealed that yeast and *C. elegans *with reduced TPI activity were short-lived. In this context, it should be noted that the TPI substrate dhap, which is greatly increased in cells with reduced TPI activity ([13,14] and see Figure 4) is thought to be a main biological source of methylglyoxal, a potent precursor of advanced glycation endproducts (AGEs) [37]. Moreover, it is feasible that the altered redox state of cells with reduced TPI activity ('reductive stress') has a negative impact on natural lifespan.

## Discussion

Here we provide the first evidence, by means of genetic and metabolic datasets along with *in silico *modeling, that active dynamic rerouting of the carbohydrate flux represents an immediate key to counteracting oxidative stress. Although earlier studies reported that an enhanced activity of the well-conserved PPP, which is strongly interconnected with the glycolytic pathway, was observed in mammalian cells under conditions of oxidative stress [7,15], the underlying cellular mechanism is far from being understood. Encouraged by the discovery that a reduction in intracellular TPI activity results in enhanced oxidant resistance in *S. cerevisiae *and *C. elegans*, we directly addressed the question of whether blockage of TPI causes a redirection of the metabolic flux from glycolysis to the PPP. By genetic means, we showed that the oxidant resistant phenotype of cells with reduced TPI activity is based on the activity of the PPP; this effect is absent in yeast cells in which the first and rate-limiting step of the PPP is inhibited. In addition, our metabolic datasets clearly support the idea that decreasing the cellular TPI activity leads to raised levels of PPP metabolites. We also provide experimental and *in silico *evidence that increased reduction of NADP^+ ^to NADPH within the PPP, which raises the electrochemical potential of the cell, is responsible for the enhanced oxidant tolerance.

Because ROS provoke a shift of the cellular redox state, which is often defined as the balance of the overall NADH/NAD^+ ^and NADPH/NADP^+^ ratios, a central task in counteracting oxidative damage is to maintain the cytoplasmic NADPH/NADP^+ ^ratio. For this process, enzymes of the PPP are crucial. In contrast to NAD(H), whose redox equivalents are shuttled between mitochondria and cytoplasm [38], the cellular pools of NADP(H) seem to be maintained independently; NADPH generated in the cytoplasm is not available to mitochondria, and vice versa. In addition, cytoplasmic and mitochondrial NADP(H) are synthesized independently from NAD(H) by different NAD and NADH kinases [39]. Moreover, the intracellular concentration of NADP(H) is low compared with that of NAD(H); it has also been reported that the majority of cellular pyridine nucleotides are found bound to protein and so a minority of the NADP(H) pool remains free [18]. Besides metabolic pathways, the cellular redox state influences cellular control tasks such as signaling and transcription, and its maintenance is therefore central to proper biological function and survival.

Although inactivation of GAPDH, the enzyme acting directly downstream of TPI, had been observed in most cell types subjected to oxidants such as hydroperoxides [5,8,9,15,27,40], the significance of this at the cellular level remained unclear. It has been speculated that GAPDH inactivation might result in a redirection of the carbohydrate flux [6,8]; however, no direct evidence for this had been presented. Here, we were able to address this question by combining genetic and metabolic analyses. Our model system is based on the fact that diamide treatment, in contrast to other oxidants, does not affect GAPDH activity in yeast. Our experiments showed that blocking GAPDH activity led to similar changes in levels of PPP metabolites as observed in cells with low TPI activity. Thus, the inactivation of GAPDH functions as a cellular switch that reroutes the carbohydrate flux to maintain the cytoplasmic NADPH/NADP^+ ^equilibrium to counteract oxidative stress. In addition, it is fairly likely that the altered levels of metabolites act as an early signaling event in cell-cycle progression and control, as it has been shown that GAPDH activity is a main regulator of H_2_O_2_-induced apoptosis [41].

In general, oxidative stress contributes profoundly to the cellular aging process, as well as to a large number of genetic and infectious diseases. Therefore, understanding the mechanisms that counteract the cellular consequences of oxidative stress is of immense interest, in particular in the perspective that enhancing cellular tolerance of eukaryotic cells to oxidative stress may result in the identification of proteins exploitable as therapeutic targets. In this light, the glucose analog and glycolytic inhibitor 2-deoxy-D-glucose (2DG) is in clinical trials as an anti-cancer therapeutic (reviewed in [42]), and was recently shown to have potent anti-epileptic properties [43]. Of note, 2DG seems to inhibit glycolysis mainly by interfering with the enzyme phosphoglucose isomerase [44,45]. Thus, it is conceivable that 2DG induces a deviation of the carbohydrate flux similar to that we have demonstrated in this study. Since epilepsy is strongly associated with oxidative stress (for review see [46]), 2DG and other glycolytic inhibitors may have promising potential as therapeutics for oxidative stress-related neuronal disorders, such as Alzheimer's disease, Parkinson's disease and trinucleotide expansion disorders.

## Materials and methods

### Plasmids

The plasmid encoding *K. lactis GDP1 *(p1696) and the p416GPD-based plasmids encoding wild-type human TPI or TPI_Ile170Val _were described earlier [11,47]. The generation of additional plasmids used in this study is described in Additional data file 4.

### Yeast cultivation and strains

Yeast was cultivated in YPD medium or synthetic complete (SC) medium lacking the indicated amino acids/bases and containing 2% glucose as described [11]. All yeast strains generated and used in this study are listed in Table 1.

**Table 1:** Yeast strains used in the study

Name	Genotype, chromosomal	Genotype, extrachromosomal	Parent strain	Reference
BY4741	*Mat *a; *his3*Δ1; *leu2*Δ0; *met15*Δ0; *ura3*Δ0		S288c	[48]
Y2546	*Mat *a; *his3*-11,15; *leu2-*3,112; *trp1-*1; *ura3-*1; *can1-*100		W303	J. Broach
MR100	*Mat *a; *his3*Δ1; *leu2*Δ0; *met15*Δ0; *ura3*Δ0; *tpi1::LEU2*		BY4741	[11]
MR101	*Mat *a; *his3*Δ1; *leu2*Δ0; *met15*Δ0; *ura3*Δ0; *tpi1::LEU2*	CEN-*URA3*-*GPDpr-hTPI*	MR100	[11]
MR105	*Mat *a; *his3*Δ1; *leu2*Δ; *met15*Δ0; *ura3*Δ0; *tpi1::LEU2*	CEN-*URA3*-*GPDpr*-*hTPI*_*Ile170Val*_	MR100	[11]
MR120	*Mat *a; *his3*Δ1; *leu2*Δ0; *met15*Δ0; *ura3*Δ0; *tpi1::LEU2 sol3::MET15*	CEN-*URA3*-*GPDpr*-*hTPI*	MR101	This study
MR121	*Mat *a; *his3*Δ1; *leu2*Δ0; *met15*Δ0; *ura3*Δ0; *tpi1::LEU2 sol4::MET15*	CEN-*URA3*-*GPDpr*-*hTPI*	MR101	This study
MR123	*Mat *a; *his3*Δ1; *leu2*Δ0; *met15*Δ0; *ura3*Δ0; *tpi1::LEU2 zwf1::KanMX4*	CEN-*URA3*-*GPDpr*-*hTPI*	MR101	This study
MR130	*Mat *a; *his3*Δ1; *leu2*Δ0; *met15*Δ0; *ura3*Δ0; *tpi1::LEU2*	CEN-*HIS3*-*GPDpr*-*hTPI*	MR101	This study
MR131	*Mat *a; *his3*Δ1; *leu2*Δ0; *met15*Δ0; *ura3*Δ0; *tpi1::LEU2*	CEN-*HIS3*-*GPDpr*-*hTPI*_*Ile170Val*_	MR101	This study
MR132	*Mat *a; *his3*Δ1; *leu2*Δ0; *met15*Δ0; *ura3*Δ0; *tpi1::LEU2 sol3::MET15*	CEN-*HIS3*-*GPDpr*-*hTPI*	MR120	This study
MR133	*Mat *a; *his3*Δ1; *leu2*Δ0; *met15*Δ0; *ura3*Δ0; *tpi1::LEU2 sol3::MET15*	CEN-*HIS3*-*GPDpr*-*hTPI*_*Ile170Val*_	MR120	This study
MR134	*Mat *a; *his3*Δ1; *leu2*Δ0; *met15*Δ0; *ura3*Δ0; *tpi1::LEU2 sol4::MET15*	CEN-*HIS3*-*GPDpr*-*hTPI*	MR121	This study
MR135	*Mat *a; *his3*Δ1; *leu2 *0; *met15*Δ0; *ura3*Δ0; *tpi1::LEU2 sol4::MET15*	CEN-*HIS3*-*GPDpr*-*hTPI*_*Ile170Val*_	MR121	This study
MR136	*Mat *a; *his3*Δ1; *leu2*Δ0; *met15*Δ0; *ura3*Δ0; *tpi1::LEU2 zwf1::KanMX4*	CEN-*HIS3*-*GPDpr*-*hTPI*	MR123	This study
MR137	*Mat *a; *his3*Δ1; *leu2*Δ0; *met15*Δ0; *ura3*Δ0; *tpi1::LEU2 zwf1::KanMX4*	CEN-*HIS3*-*GPDpr*-*hTPI*_*Ile170Val*_	MR123	This study

The deletion strains Δ*tpi1*Δ*zwf1 *(MR123), Δ*tpi1*Δ*sol3 *(MR120) and Δ*tpi1*Δ*sol4 *(MR121) were generated by single gene replacement approaches using the *kanMX4 *marker in case of the *ZWF1 *gene deletion or *MET15 *in case of the *SOL3 *and *SOL4 *gene deletion. Briefly, PCR products encoding the *MET15 *gene or the *kanMX4 *gene were amplified by PCR using plasmids pRS411 [48] or pUG6 [49] as a template. The respective primer pairs encompassing a homologous boundary 5' and 3' to the target locus are listed in Additional data file 4. After transformation of the parental Δ*tpi1 *strain MR101, single recombinants were selected on synthetic minimal media supplemented with histidine or complete media (YPD) containing 200 μg/ml G418 (Gibco, Invitrogen, Carlsbad, CA). After selection and validation of the respective clones, the newly generated strains were transformed with p413GPD-based centromeric plasmids encoding human TPI or TPI_Ile170Val_. Subsequently, single clones were isolated on SC^-his ^media, and counterselected against the URA3-CEN plasmid on SC^-his ^media containing 0.15% 5-fluoroorotic acid.

For the oxidative stress resistance experiments, yeast cells were grown overnight in SC medium lacking the amino acids or bases as indicated, diluted to the same optical density at 600 nm and spotted as fivefold dilution series onto agar plates supplemented with differing concentrations of diamide (in 0.2 mM steps) or H_2_O_2 _(in 0.05 mM steps). Using liquid cultures, oxidative stress was induced by adding 2 mM H_2_O_2 _to exponentially growing cultures for 30 min as described earlier [28].

Yeast median replicative lifespan was assayed by microdissection of a cohort of at least 40 cells per strain on defined SC medium as described earlier [50]. To determine whether two given lifespan distributions were significantly different at the 95% confidence level, Breslow, Tarone-Ware and log-rank statistics were used, and statistical calculations were performed using the SPSS 13.0 software package.

### SDS-PAGE and western blotting

SDS-PAGE and western blotting were carried out as described previously [11] using a BioRad Mini Protean gel chamber and a semidry electroblotter. Primary antibodies were used in the following dilutions: anti-TPI (1:4000 [51]), anti-G6PDH (1:5000, Sigma Aldrich A9521), anti-HSP82 (cross-reacts with *C. elegans daf21*, 1:4000, provided by Susan Lindquist) and polyclonal anti-GAPDH (1:2500, Abcam 36840-1).

### Enzyme activity assays

GAPDH activity assays were performed with minor modifications as described [52]. Briefly, cell lysates were added to a photometric cuvette containing 1 ml of 1 mM NAD^+ ^dissolved in 30 mM Na-pyrophosphate buffer, pH 8.4. The reaction was started at room temperature by adding 10 μl 40 mM gly3p. Enzyme activity under steady-state conditions was calculated as the rate of NAD^+ ^reduction per minute determined in 5–10 sec intervals as the absorbance of NADH at a wavelength of 340 nm using an Amersham Ultrospec 3100 spectrophotometer. TPI activity of whole-cell extracts was determined as described previously [11].

### *C. elegans *culture and assays

Nematodes were cultured at 20°C on NG agar plates with the *E. coli *strain OP50. We used the strains N2 and *daf-2*(*e1370*). RNAi experiments were carried out on NGM agar plates supplemented with 50 μg/ml ampicillin and 1 mM isopropyl-beta-D-thiogalactopyranoside. Overnight bacterial cultures (LB medium with 100 μg/ml ampicillin) of RNAi-producing *E. coli *from the RNAi library [53] (Geneservice, Cambridge, UK) were concentrated by half and seeded on the plates, dried overnight at room temperature and then kept at 4°C for subsequent use.

*C. elegans *lifespan assays were performed at 20°C. Wild-type N2 worms were fed with *E. coli *that produce double-stranded RNA of the *C. elegans tpi-1 *gene (Y17G7B.7; MRC Geneservice_Location: II-8I11) or contain the empty RNAi vector L4440 as control. Twenty-five L4 worms of the F1 generation were set up onto one RNAi plate. During the reproductive period the worms were transferred daily; later on every week. The RNAi plates were not older than 4 days and were seeded one or two days before use. The number of dead versus live animals was determined every day. Day 0 corresponds to the L4 stage. *P *values were calculated on the pooled data of all of the experiments done in each set by using the log-rank (Mantel-Cox) [54].

Oxidative stress resistance was assayed by transferring 1-day-old adults on plates containing 250 mM diamide (Sigma) or 10 μM juglone (Sigma). Survival was scored over a 10-h period at 20°C. Diamide and juglone plates were produced one day before use and experiments were repeated at least twice. *p *values were calculated by using the log-rank (Mante-Cox) method.

### Quantitative metabolite measurements

For the measurements of sugar phosphates, yeast cultures were grown in rich medium (YPD) overnight. Subsequently, cell cultures were diluted to an OD_600 _of 0.15, and two yeast cultures of each overnight culture were grown in parallel to mid-log phase. Cells were then collected by centrifugation, washed, shock-frozen in liquid nitrogen and lysed by glass beads in cold Hank's Balanced Salt Solution (without phenol red) containing 2% perchloric acid for immediate denaturation of proteins after lysis. All steps of the lysate generation were carried out in a cold room at 4°C. Samples were then stored at -80°C, thawed and 50 μl internal standard (10 μM ^13^C_6_-glucose-6-phosphate) was added to 50 μl of the lysate. Samples were then neutralized with 1 M phosphate buffer (pH 11.5) and centrifuged for 5 min at 21,000 *g *at 4°C. Supernatants were transferred to glass vials and capped. Calibrators of dhap, r5p, x5p, g6p, s7p, 6pg, gly3p and gol3p were included in each batch of samples and were processed as described above. LC-MS/MS analysis for quantification of metabolites was carried out as described earlier [55]. Briefly, liquid chromatography was performed using a Perkin-Elmer series 200 pump with a 3.9 × 150 mm Symmetry C_18 _HPLC column (bead size 5 μm, Waters Chromatography, Etten-Leur, The Netherlands). For gradient elution, a binary solvent was used as described before [55]. Solvent A consisted of 12.5% acetonitrile (ACN)/water containing 500 mg/l octylammonium acetate (pH 7.5) and solvent B consisted of 50% ACN/water containing 500 mg/l octylammonium acetate (pH 7.5). The column was rinsed with solvent A for 3 min to load the column with ion-pairs. The initial composition of the binary solvent was 100% A, followed by a linear gradient to 40% A and 60% B in 8 min. Thereafter, the mobile-phase composition was changed to 100% B and stayed there for 2 min. Finally the mobile-phase composition was changed to 100% A for 5 min to reload the column with ion-pairs. The flow rate was set to 1 ml/min and was split post-column into a ratio of 1:4, resulting in an inlet flow into the tandem mass spectrometer of 200 μl/min; 3 μl sample was injected onto the column and the total run time was 20 min.

Detection of the sugar phosphates was carried out on an API-3000 tandem mass spectrometer (PE-Sciex, Applied Biosystems, Foster City, CA) equipped with an electron ion spray source (Turbo Ion Spray, Applied Biosystems) operating in negative multiple reaction monitoring (MRM-mode). The MRM transitions (Q1/Q3) settings for the different sugar phosphates were: dhap/Gly3p, m/z -169/-97; gol3p, m/z -171/-79; r5p and x5p, m/z -229/-97; g6p, m/z -259/-97; ^13^C_6_-g6p (internal standard): m/z -265/-97; 6pg: m/z -275/-97, and s7p, m/z -289/-97. Data were acquired and processed using Analyst™ for Windows NT software (Ver. 1.3.1). To convert the quantitative measurements into estimated, absolute cellular metabolite concentrations (required for parameter fitting of the mathematical model), we used a dhap concentration of 0.76 mM, which was recently determined enzymatically in mid-log phase wild-type yeast cells [14], as a base to calculate a calibration factor of 0.477. Absolute as well as relative metabolite concentrations are given in Additional data file 3.

To determine the cellular NADPH/NADP^+ ^concentration, yeast was grown in YPD to mid-log phase, collected by centrifugation and washed in Tris-EDTA buffer. Afterwards, the cell pellet was shock-frozen, thawed, and resuspended in TE buffer. Cells were lysed by rigorous mixing (3 × 2 min, 4°C) using acid-washed glass beads before the supernatant was cleared by centrifugation. Subsequently, 34:24:1 phenol: chloroform:isoamyl-alcohol was added to the supernatant and supplemented with 6.6 mM EDTA as described by Noack *et al*. [24]. After rigorous mixing, phase separation was enforced by centrifugation and the aqueous phase was then extracted twice with water-saturated diethlyether. These extracts were immediately frozen in liquid nitrogen and stored at -80°C until LC-MS/MS analysis. The quality of the extraction method was controlled by HPLC analysis using an C_18 _(RP) column and UV detection at 254 nm (data not shown). Liquid chromatography and MS/MS detection was performed as described above for the other metabolites; the MRM transitions (Q1/Q3) settings were m/z -742.2/-620.1 for NADP^+ ^and m/z -744.2/-79.0 for NADPH. Standards were obtained from Sigma (St Louis, MO).

### Kinetic modeling

To develop a kinetic model of the combined reactions of glycolysis and the PPP, we used the model of Teusink *et al*. [31] as a basis for the glycolytic reactions (available in SBML format from JWS online at [56]). In brief, we included the reaction between dhap and gly3p as reversible Michaelis-Menten kinetics and added all the reactions belonging to the PPP. The following equations were used for the different kinetic types (V^+ ^= maximum rate of forward reaction, V^- ^= maximum rate of backward reaction, K_s _= Michaelis-Menten constant of the substrate, K_p _= Michaelis-Menten constant of the product).

Irreversible uni-uni Michaelis-Menten (MM):

$$\text{v}=\frac{{\text{V}}^{+}\cdot \text{S}}{\text{S}+\text{Ks}}$$

Reversible uni-uni MM:

$$\text{v}=\frac{{\text{V}}^{+}\cdot \frac{\text{S}}{\text{Ks}}-{\text{V}}^{-}\cdot \frac{\text{P}}{\text{Kp}}}{1+\frac{\text{S}}{\text{Ks}}+\frac{\text{P}}{\text{Kp}}}$$

Reversible bi-bi MM:

$$\text{v}=\frac{{\text{V}}^{+}\cdot \frac{\text{S}1}{\text{Ks}1}\cdot \frac{\text{S}2}{\text{Ks}2}-{\text{V}}^{-}\cdot \frac{\text{P}1}{\text{Kp}1}\cdot \frac{\text{P}2}{\text{Kp}2}}{\left(1+\frac{\text{S}1}{\text{Ks}1}+\frac{\text{P}1}{\text{Kp}1}\right)\cdot \left(1+\frac{\text{S}2}{\text{Ks}2}\cdot \frac{\text{P}2}{\text{Kp}2}\right)}$$

Irreversible bi-bi MM with inhibition by product 1:

$$\text{v}=\frac{{\text{V}}^{+}\cdot \frac{\text{S}1}{\text{Ks}1}\cdot \frac{\text{S}2}{\text{Ks}2}}{\left(1+\frac{\text{S}1}{\text{Ks}1}+\frac{\text{P}1}{\text{Kp}1}\right)\cdot \left(1+\frac{\text{S}2}{\text{Ks}2}\right)}$$

All the reactions included in the model, with the kinetic type and kinetic parameters used, are given in Additional data file 2. The type of kinetics was, in most cases, derived using information from Stryer [57]. Enzyme activity that was only provided as U/mg protein in the literature was converted to mM/min on the assumption that the typical protein concentration in the cytoplasm is around 0.26 μg/μl (J. Snoep, personal communication). Another relationship that was used to convert different units is the following constraint:

$${\text{k}}_{\text{eq}}=\frac{{\text{V}}^{+}}{{\text{V}}^{-}}\cdot \frac{{\prod \text{K}}_{\text{mProducts}}}{{\prod \text{K}}_{\text{mSubstrates}}}$$

Because almost all reactions use some form of saturation kinetics, it is possible that the system does not enter a steady state (that is, some metabolites accumulate without limit). Using the kinetic data from five different species (human, cow, rabbit, yeast, *E. coli*) that were measured over a time span of more than 30 years, this indeed happened. To avoid this non-physiological situation, a V_max _of 4 mM/min was used instead of the calculated 0.53 mM/min for reaction 17, and V_max _values of 4 and 2 mM/min were used instead of the calculated values of 0.04 and 0.02 mM/min for reaction 22 (see Additional data file 2). The model was implemented using CellDesigner 3.2 [58]. The generated SBML code was then used with Copasi 4B20 [32] to perform the parameter fitting. The SBML code of the model is available as Additional data file 5.

## Additional data files

Additional data are available with this article online. Additional data file 1 contains details of the *C. elegans *experiment. Additional data file 2 contains a figure and a table of the reactions included in the mathematical model to study the effects of a diminished TPI or GAPDH activity on the flux through glycolysis and the pentose phosphate pathway. Additional data file 3 contains tables of metabolite concentrations. Additional data file 4 contains information on the generation of plasmids and oligonucleotides used in this study. Additional data file 1 contains the SBML code for the mathematical model.

## Supplementary Material

Additional data file 1 *C. elegans* experiment. Click here for file

Additional data file 2 A figure and a table of the reactions included in the mathematical model. Click here for file

Additional data file 3 Metabolite concentrations. Click here for file

Additional data file 4 Plasmids and oligonucleotides. Click here for file

Additional data file 5 SBML code for the mathematical model. Click here for file
