# Supplementary material for: Dynamic rerouting of the carbohydrate flux is key to counteracting oxidative stress
Source: J Biol. 2007 Dec 21;6(4):10. doi: 10.1186/jbiol61 (PMC2373902; doi:10.1186/jbiol61)
Supplement: Additional data file 1 — C. elegans experiment. [file jbiol61-S1.doc]

# Additional file 1

Markus Ralser et al.

# Dynamic re-routing of the carbohydrate flux is key to counteracting oxidative stress

**“Stress and Ageing of *C. elegans*”**

| **Experiment** | **RNAi** | **Lifespan, days** | | **No. of animals (no. of exps)** | ***p* value** |
| --- | --- | --- | --- | --- | --- |
|  |  | **Mean ± SD** | **Maximum ± SD** |  |  |
| **Lifespan** | vector | 15.7 ± 0.9 | 23.0 ± 0 | 117 (2) |  |
|  | *tpi-1* | 14.4 ± 0.9 | 21.0 ± 1.4 | 80 (2) | 0.0016 |
|  | vector ^1^ | 16.2 | 23 | 74 (1) |  |
|  | *tpi-1*^1^ | 14.8 | 21 | 47 (1) | 0.0035 |
| 10 μM juglone | vector | 4.2 ± 0.8 | 5.7 ± 1.2 | 64 (3) |  |
|  | *tpi-1* | 5.5 ± 0.4 | 7.7 ± 0.6 | 64 (3) | 1.13e-07 |
| 250 mM diamide | vector | 7.5 ± 0.3 | 10.5 ± 0.7 | 65 (2) |  |
|  | *tpi-1* | 8.6 ± 0.3 | 11.0 ± 0 | 55 (2) | 0.011 |

^1^young adults were set up directly on RNAi plates.

Wild type N2 worms were grown one generation before the experiment and during the experiment on different RNAi strains, exceptions noted.

SDs were calculated on the average of the mean or maximum of individual experiments. Significance tested against L4440 control. *P* values were determined on pooled experiments using the log rank test (Mantel-Cox).
