# Supplementary material for: Dynamic rerouting of the carbohydrate flux is key to counteracting oxidative stress
Source: J Biol. 2007 Dec 21;6(4):10. doi: 10.1186/jbiol61 (PMC2373902; doi:10.1186/jbiol61)
Supplement: Additional data file 2 — A figure and a table of the reactions included in the mathematical model. [file jbiol61-S2.doc]

# Additional data file 2

Markus Ralser et al.

# Dynamic re-routing of the carbohydrate flux is key to counteracting oxidative stress

“**Mathematic al Model of Glycolysis and the Pentose Phosphate Pathway**”


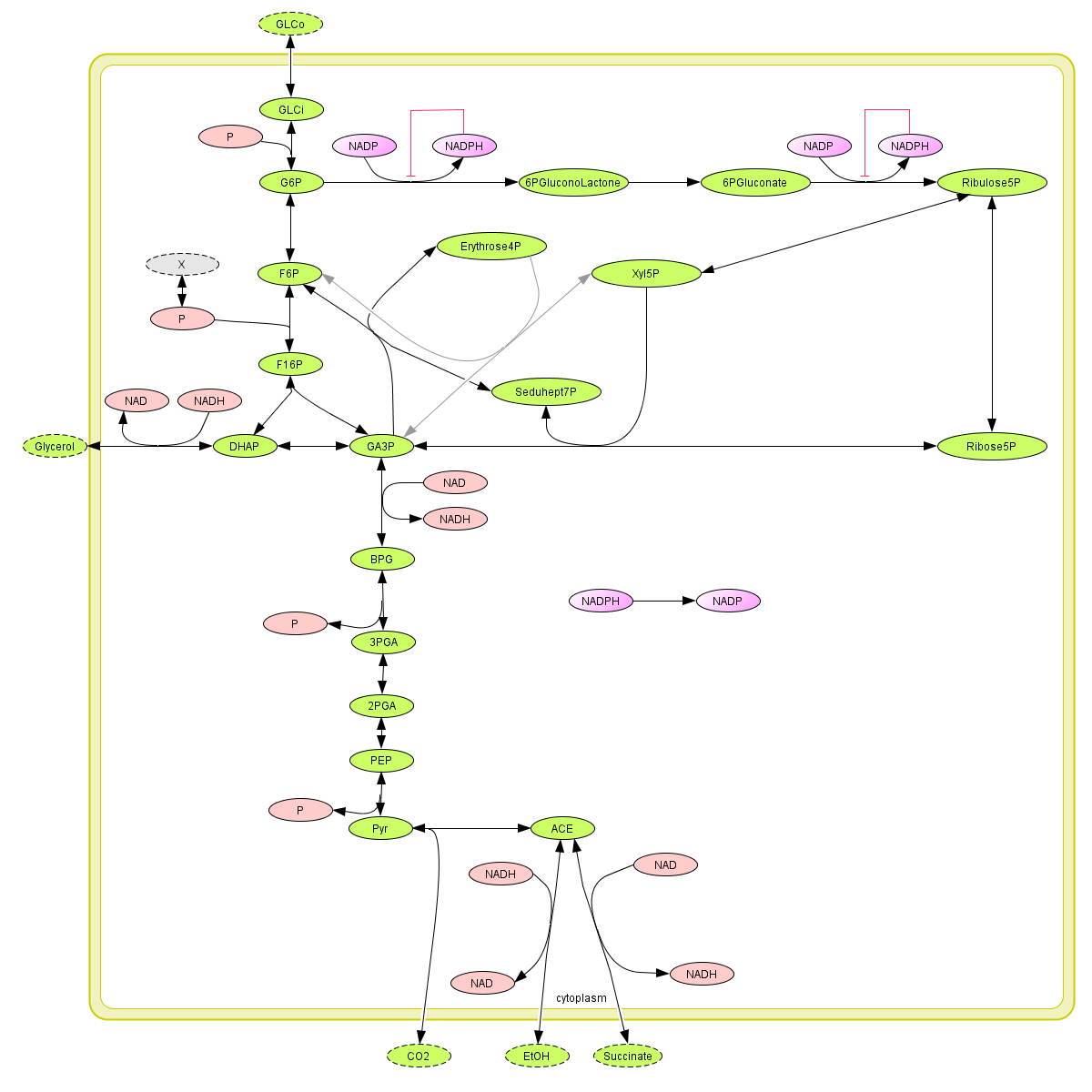


**Supplementary Figure 1:**

Reactions included in the mathematical model to study the effects of a diminished TPI or GAPDH activity on the flux through glycolysis and the pentose phosphate pathway. Substances with a dashed outline are assumed to have a constant concentration; “P” indicates the generation respectively consumption of high-energy phosphates and thin red lines indicate a product inhibition.

**Table:** List of biochemical reactions included in the mathematical model, together with the used kinetic type and the numerical values for the kinetic parameters. The glycolytic reactions are mostly based on the model of Teusink *et al.* .

|  | Reaction | Kinetic type | Kinetic parameters | Ref. |
| --- | --- | --- | --- | --- |
| 1 | GLCo ↔ GLCi | custom type | see [1] | [1] |
| 2 | GLCi + P ↔ G6P | rev. bi-bi | KmGLCi = 0.08 mM  KmATP = 0.15 mM  KmG6P = 30 mM  KmADP = 0.23 mM | [1] |
| 3 | G6P ↔ F6P | rev. uni-uni | KmG6P = 1.4 mM  KmF6P = 0.3 mM | [1] |
| 4 | F6P +P ↔ F16P | custom type | see [1] | [1] |
| 5 | P ↔ X | custom type | see [1] | [1] |
| 6 | F16P ↔ DHAP + GA3P | ordered uni-bi (eqn. A4) | KmF16P = 0.3 mM  KmDHAP = 2.4 mM  KmGA3P = 2 mM  KiGA3P = 10 mM | [1] |
| 7 | DHAP ↔ GA3P | rev. uni-uni | KmGA3P = 1.27 mM  KmDHAP = 1.23 mM  V→DHAP = 10900 mM/min  V→GA3P = 555 mM/min | [2] |
| 8 | DHAP+NADH ↔ Glycerol + NAD | rev. bi-bi | KmDHAP = 0.4 mM  KmNADH = 0.023 mM  KmNAD = 0.93 mM  KmG3P = 1 mM  [1] | [1] |
| 9 | GA3P + NAD ↔ BPG + NADH | rev. bi-bi | KmGA3P = 0.21 mM  KmNAD = 0.09 mM  KmBPG = 9.8*10^-3^ mM  KmNADH = 0.06 mM  [1] | [1] |
| 10 | BPG ↔ 3PGA + P | rev. bi-bi | KmBPG = 0.003 mM  KmADP = 0.2 mM  KmATP = 0.3 mM  Km3PGA = 0.53 mM | [1] |
| 11 | 3PGA ↔ 2PGA | rev. uni-uni | Km3PGA = 1.2 mM  Km2PGA = 0.1 mM | [1] |
| 12 | 2PGA ↔ PEP | rev. uni-uni | Km2PGA = 0.04 mM  KmPEP = 0.5 mM | [1] |
| 13 | PEP ↔ Pyr + P | rev. bi-bi | KmPEP = 0.15 mM  KmADP = 0.53 mM  KmATP = 1.5 mM  KmPyr = 21 mM | [1] |
| 14 | Pyr ↔ ACE + CO_2_ | irrev. Hill | KmPyr = 4.33 mM  HillCoeff = 1.9 | [1] |
| 15 | ACE + NADH ↔ EtOH + NAD | ordered bi-bi | KmEtOH = 17 mM  KmNAD = 0.17 mM  KmNADH = 0.11 mM  KmACE = 1.11 mM  KiEtOH = 90 mM  KiNAD = 0.92 mM  KiNADH = 0.031 mM  KiACE = 1.1 mM | [1] |
| 16 | ACE + NAD ↔ Succinate + NADH | mass action | see [1] | [1] |
| 17 | G6P + NADP ↔ 6PGluconoLactone + NADPH | irrev. bi-bi with product inhibition | Vmax = 4 mM/min  KmG6P = 0.04 mM  KmNADP = 0.02 mM  KiNADPH = 0.017 mM | [3] |
| 18 | 6PGluconoLactone → 6PGluconate | irrev. uni-uni | Vmax = 4 mM/min  Km6PGl = 0.8 mM | [4] |
| 19 | 6PGluconate + NADP ↔ Ribulose5P + NADPH | irrev. bi-bi with product inhibition | Vmax = 4 mM/min  KmGluconate = 0.02 mM  KmNADP = 0.03 mM  KiNADPH = 0.03 mM | [5] |
| 20 | Ribulose5P ↔ Ribose5P | rev. uni-uni | V→Ribu = 3458 mM/min  V→Ribo = 3458 mM/min  KmRibu = 1.6 mM  KmRibo = 1.6 mM | [6] |
| 21 | Ribulose5P ↔ Xyl5P | rev. uni-uni | V→Xyl = 1039 mM/min  V→Ribu = 1039 mM/min  KmXyl = 1.5 mM  KmRibu = 1.5 mM | [7] |
| 22 | Ribose5P + Xyl5P ↔ GA3P + Seduhept7P | rev. bi-bi | V→GA3P = 4 mM/min  V→Xyl = 2 mM/min  KmRibo = 0.1 mM  KmXyl = 0.15 mM  KmGA3P = 0.1 mM  KmS7P = 0.15 mM | [8, 9] |
| 23 | Seduhept7P + GA3P ↔ F6P + Erythrose4P | rev. bi-bi | V→F6P = 55 mM/min  V→S7P = 10 mM/min  KmS7P = 0.18 mM  KmGA3P = 0.22 mM  KmF6P = 0.32 mM  KmEry = 0.018 mM | [8, 10, 11] |
| 24 | Erythrose4P + Xyl5P ↔ GA3P + F6P | rev. bi-bi | V→F6P = 3.2 mM/min  V→Xyl = 43 mM/min  KmXyl = 0.16 mM  KmEry = 0.09 mM  KmGA3P = 2.1 mM  KmF6P = 1.1 mM | [12-14] |
| 25 | NADPH → NADP | irrev. mass action | k_NADPH_ = 2 min^-1^ |  |

**References**

1. Teusink B, Passarge J, Reijenga CA, Esgalhado E, van der Weijden CC, Schepper M, Walsh MC, Bakker BM, van Dam K, Westerhoff HV, Snoep JL: **Can yeast glycolysis be understood in terms of in vitro kinetics of the constituent enzymes? Testing biochemistry.** *Eur J Biochem* 2000, **267:**5313-5329.

2. Krietsch WK: **Triosephosphate isomerase from yeast.** *Methods Enzymol* 1975, **41:**434-438.

3. Aksoy Y, Ogus IH, Oauzer N: **Purification and some properties of human placental glucose-6-phosphate dehydrogenase.** *Protein Expr Purif* 2001, **21:**286-292.

4. Bauer HP, Srihari T, Jochims JC, Hofer HW: **6-phosphogluconolactonase. Purification, properties and activities in various tissues.** *Eur J Biochem* 1983, **133:**163-168.

5. Pearse BM, Rosemeyer MA: **Human 6-phosphogluconate dehydrogenase. Purification of the erythrocyte enzyme and the influence of ions and NADPH on its activity.** *Eur J Biochem* 1974, **42:**213-223.

6. Reuter R, Naumann M, Bar J, Miosga T, Kopperschlager G: **Ribose-5-phosphate isomerase from Saccharomyces cerevisiae: purification and molecular analysis of the enzyme.** *Bioseparation* 1998, **7:**107-115.

7. Bar J, Naumann M, Reuter R, Kopperschlager G: **Improved purification of ribulose 5-phosphate 3-epimerase from Saccharomyces cerevisiae and characterization of the enzyme.** *Bioseparation* 1996, **6:**233-241.

8. Goldberg RN, Tewari YB, Bhat TN: **Thermodynamics of enzyme-catalyzed reactions--a database for quantitative biochemistry.** *Bioinformatics* 2004, **20:**2874-2877.

9. Takeuchi T, Nishino K, Itokawa Y: **Purification and characterization of, and preparation of an antibody to, transketolase from human red blood cells.** *Biochim Biophys Acta* 1986, **872:**24-32.

10. Kuhn E, Brand K: **Purification and properties of transaldolase from bovine mammary gland.** *Biochemistry* 1972, **11:**1767-1772.

11. Tsolas O, Horecker BL: In *The Enzymes.* P. D. Boyer, ed.; 1972: 259-280

12. Datta AG, Racker E: **Mechanism of action of transketolase. I. Properties of the crystalline yeast enzyme.** *J Biol Chem* 1961, **236:**617-623.

13. Masri SW, Ali M, Gubler CJ: **Isolation of transketolase from rabbit liver and comparison of some of its kinetic properties with transketolase from other sources.** *Comp Biochem Physiol B* 1988, **90:**167-172.

14. Sprenger GA, Schorken U, Sprenger G, Sahm H: **Transketolase A of Escherichia coli K12. Purification and properties of the enzyme from recombinant strains.** *Eur J Biochem* 1995, **230:**525-532.
