# Supplementary material for: Dynamic rerouting of the carbohydrate flux is key to counteracting oxidative stress
Source: J Biol. 2007 Dec 21;6(4):10. doi: 10.1186/jbiol61 (PMC2373902; doi:10.1186/jbiol61)
Supplement: Additional data file 3 — Metabolite concentrations. [file jbiol61-S3.pdf]

Additional file 3

Markus Ralser et al.,
Dynamic re-routing of the carbohydrate flux is key to counteracting oxidative stress

„Quantification of Yeast Carbohydrate Metabolites"

Dihydroxyacetone phosphate (dhap)

Strain and condition	*Measured concentration* (nmol/ml*OD$_{600}$)	*SD*	*Calculated biological concentration* (mMol)
BY4741	1,59	0,16	0,76
BY4741 with H_2O_2	2,36	0,20	1,13
MR101 (70% TPI activity)	1,99	0,21	0,95
MR105 (20% TPI activity)	2,60	0,11	1,24

Glyceraldehyde-3-phosphate (gly3p)

Strain and condition	*Measured concentration* (nmol/ml*OD$_{600}$)	*SD*	*Calculated biological concentration* (mMol)
BY4741	0,12	0,03	0,06
BY4741 with H_2O_2	0,19	0,02	0,09
MR101 (70% TPI activity)	0,07	0,01	0,04
MR105 (20% TPI activity)	0,08	0,02	0,04

Glucose-6-phosphate and Fructose-6-phosphate (measured as the sum of both) (g6p)

Strain and condition	*Measured concentration* (nmol/ml*OD$_{600}$)	*SD*	*Calculated biological concentration* (mMol)
BY4741	1,32	0,08	0,63
BY4741 with H_2O_2	2,02	0,12	0,96
MR101 (70% TPI activity)	1,69	0,29	0,80
MR105 (20% TPI activity)	2,93	0,31	1,40

Ribose-5-phosphate (r5p)

Strain and condition	*Measured concentration* (nmol/ml*OD$_{600}$)	*SD*	*Calculated biological concentration* (mMol)
BY4741	1,44	0,07	0,69
BY4741 with H_2O_2	2,84	0,06	1,36
MR101 (70% TPI activity)	1,52	0,08	0,73
MR105 (20% TPI activity)	2,08	0,30	0,99

Sedoheptulose-7-Phosphate (s7p)

Strain and condition	*Measured concentration* (nmol/ml*OD$_{600}$)	*SD*	*Calculated biological concentration* (mMol)
BY4741	1,41	0,12	0,67
BY4741 with H_2O_2	35,68	2,49	17,02
MR101 (70% TPI activity)	1,10	0,12	0,52
MR105 (20% TPI activity)	1,61	0,27	0,77

Glycerol-3-phosphate (gol3p)

Strain and condition	*Measured concentration* (nmol/ml*OD_{600})	*SD*	*Calculated biological concentration* (mMol)
BY4741	0,81	0,09	0,39
BY4741 with H_2O_2	0,69	0,15	0,33
MR101 (70% TPI activity)	0,57	0,02	0,27
MR105 (20% TPI activity)	0,57	0,19	0,27

6-Phosphogluconate (6pg)

Strain and condition	*Measured concentration* (nmol/ml*OD_{600})	*SD*	*Calculated biological concentration* (mMol)
BY4741	0,03	0,003	0,02
BY4741 with H_2O_2	0,21	0,02	0,10
MR101 (70% TPI activity)	0,07	0,005	0,03
MR105 (20% TPI activity)	0,12	0,02	0,06

Xylulose-5-phosphate and Ribulose-5-phosphate (measured as the sum of both) (x5p)

Strain and condition	*Measured concentration* (nmol/ml*OD_{600})	*SD*	*Calculated biological concentration* (mMol)
BY4741	0,49	0,03	0,23
BY4741 with H_2O_2	3,90	0,16	1,86
MR101 (70% TPI activity)	0,58	0,06	0,28
MR105 (20% TPI activity)	0,98	0,15	0,47

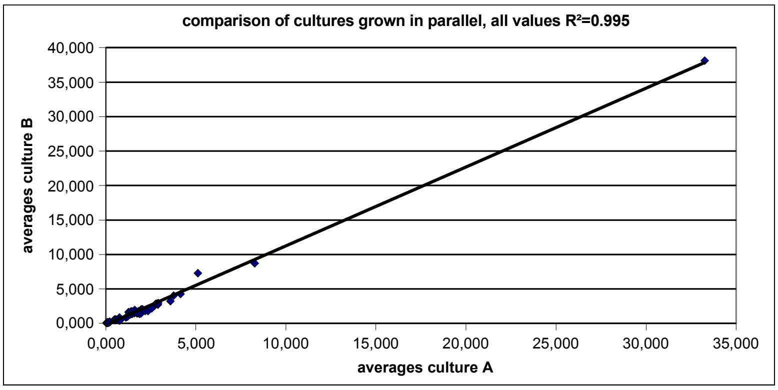

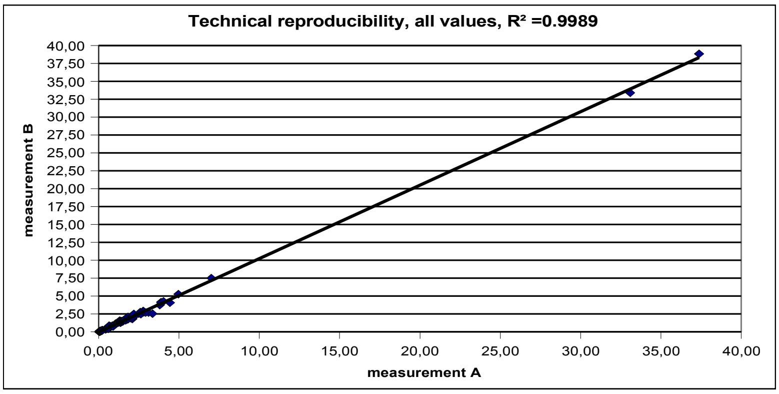

(Upper panel) For analyzing the biological reproducibility, the metabolite concentrations were measured from cultures grown in parallel.
(Lower panel) For quality control of the metabolite quantifications and for analyzing the technical reproducibility, each metabolite was measured in duplicate.
