# Supplementary material for: Dynamic rerouting of the carbohydrate flux is key to counteracting oxidative stress
Source: J Biol. 2007 Dec 21;6(4):10. doi: 10.1186/jbiol61 (PMC2373902; doi:10.1186/jbiol61)
Supplement: Additional data file 4 — Plasmids and oligonucleotides. [file jbiol61-S4.doc]

# Additional data file 4

Markus Ralser et al.

# Dynamic re-routing of the carbohydrate flux is key to counteracting oxidative stress

**Plasmids**

p416GPD-TPI-WT and p416GPD-TPI-Ile170Val encoding human wild-type or mutant TPI were described earlier [1]. Plasmids p413GPD-TPI-WT and p413GPD-TPI-Ile170Val were generated by treating the respective plasmids with *Bam*HI and *Xho*I, and the the TPI encoding DNA fragment was subcloned into the respective sites of the CEN plasmid p413GPD [2]. Moreover, plasmids for the expression of human TPI under the control of the *CYC1* or *TEF1* promoters were generated by subcloning the respective DNA fragments isolated from p416GPD-TPI-WT into the *Bam*HI/*Xho*I sites of p416TEF (Acc.No. EF210199) or the *BamH*I/*Sal*I sites of p416CYC (Acc.No. EF210198), respectively. p416GPD-yeast TPI was generated by amplifying the *TPI1* coding sequence (YDR050C) using oligonucleotides GAGGATCCATGGCTAGAACTTTCTTTGT and GAACTCGAGTTAGTTTCTAGA GTTGATGA from *S. cerevisiae* genomic DNA. Subsequently, the resultant PCR fragment was treated with *BamH*I/*Xho*I and ligated into the respective sites of the vector p416GPD (Acc.No. DQ269148).

For the generation of plasmids p423GPD-*EcoGAP*, the CDS of the *EcoGAP* was amplified from *E. coli* genomic DNA isolated from the strain *Xl1-blue* [3] using oligonucleotides GAGGATCCATGACTATCAAAGTAGGTAT and CATCTCGAGTTATTTGGAGA TGTGAGCGA. Afterwards, the DNA fragment was treated with *Bam*HI/*Xho*I and ligated into the 2µ vector p423GPD [2], respectively. Yeast *TDH3* (*YGR192C)* was amplified using primer pairs GAGGATCCATGGTTAGAGTTGCTATTAACGGTTTC and CAGCTC GAGTTAAGCCTTGGCAACGTGTT, the resulting fragment was treated with *Bam*H1/*Xho*1 and ligated into the *Bam*H1/*Xho*1 sites of p423GPD.

The *K. lactis GDP1* (p1696) expression plasmid was kindly provided by Peter Richard (VTT Biotech, Finland) and described earlier [4].

Underlined sequences represent introduced restriction sites. PCR reactions were performed as described earlier [5], and all PCR fragments generated were verified by sequencing.

**Oligonucleotides**

For generating the yeast strains described in this study the following oligonucleotides were used:

a) MR120

*MET15::sol3-s* atagtaaagtatattttctttgcgcggtgttataatttgctctttgcagattgtactgagagtgcacc

and *MET15::sol3*-as gtaatattagcatctgaggttcgaaagctcgcatgtgtgtcttttTCATGGTTTTTGGCCA

b) MR121

*MET15:*:*sol4*-s caaagaatgccattcatcaaataatccacaaccacctcaagaaaaATGCCATCTCATTTCGAT

*and MET15::sol4*-as tctgtcgtgattaacggagccagtttcgtataaagacgagtgtaaTCATGGTTTTTGGCCA

c) MR123

*KanMX::zwf1*-s acagaaagagtaaatccaatagaatagaaaaccacataaggcaagATGGGTAAGGAAAAGACTCA

and *KanMX::zwf1-*as aaaatttcagtgacttagccgataaatgaatgtgcttgcatttttTTAGAAAAACTCATCGAGCA

**References**

1. Ralser M, Heeren G, Breitenbach M, Lehrach H, Krobitsch S: **Triose Phosphate Isomerase Deficiency Is Caused by Altered Dimerization-Not Catalytic Inactivity-of the Mutant Enzymes.** *PLoS ONE* 2006, **1:**e30.

2. Mumberg D, Muller R, Funk M: **Yeast vectors for the controlled expression of heterologous proteins in different genetic backgrounds.** *Gene* 1995, **156:**119-122.

3. Bullock WO, Fernandez JM, Short JM: **Xl1-Blue - a High-Efficiency Plasmid Transforming Reca Escherichia-Coli Strain with Beta-Galactosidase Selection.** *Biotechniques* 1987, **5:**376-&.

4. Verho R, Londesborough J, Penttila M, Richard P: **Engineering redox cofactor regeneration for improved pentose fermentation in Saccharomyces cerevisiae.** *Appl Environ Microbiol* 2003, **69:**5892-5897.

5. Ralser M, Querfurth R, Warnatz HJ, Lehrach H, Yaspo ML, Krobitsch S: **An efficient and economic enhancer mix for PCR.** *Biochem Biophys Res Commun* 2006, **347:**747-751.
